# Recent advances in carrageenan-based films for food packaging applications

**DOI:** 10.3389/fnut.2022.1004588

**Published:** 2022-09-09

**Authors:** Cheng Cheng, Shuai Chen, Jiaqi Su, Ming Zhu, Mingrui Zhou, Tianming Chen, Yahong Han

**Affiliations:** ^1^Key Laboratory of Aquaculture Facilities Engineering, Ministry of Agriculture and Rural Affairs, College of Engineering, Huazhong Agricultural University, Wuhan, China; ^2^School of Public Health, Wuhan University, Wuhan, China; ^3^Beijing Advanced Innovation Center for Food Nutrition and Human Health, Key Laboratory of Functional Dairy, Ministry of Education, College of Food Science and Nutritional Engineering, China Agricultural University, Beijing, China

**Keywords:** carrageenan, food packaging, film-forming methods, film properties, formation mechanism

## Abstract

In order to solve the increasingly serious environmental problems caused by plastic-based packaging, carrageenan-based films are drawing much attentions in food packaging applications, due to low cost, biodegradability, compatibility, and film-forming property. The purpose of this article is to present a comprehensive review of recent developments in carrageenan-based films, including fabrication strategies, physical and chemical properties and novel food packaging applications. Carrageenan can be extracted from red algae mainly by hydrolysis, ultrasonic-assisted and microwave-assisted extraction, and the combination of multiple extraction methods will be future trends in carrageenan extraction methods. Carrageenan can form homogeneous film-forming solutions and fabricate films mainly by direct coating, solvent casting and electrospinning, and mechanism of film formation was discussed in detail. Due to the inherent limitations of the pure carrageenan film, physical and chemical properties of carrageenan films were enhanced by incorporation with other compounds. Therefore, carrageenan-based films can be widely used for extending the shelf life of food and monitoring the food freshness by inhibiting microbial growth, reducing moisture loss and the respiration, etc. This article will provide useful guidelines for further research on carrageenan-based films.

## Introduction

Food packaging is to separate food items from the surrounding environment, preventing microorganisms, oxygen, and water from contacting food, thus ensuring food quality and extending food shelf life (1, 2). It is estimated that approximately 36% of food packaging materials are petroleum-based plastics, including polyethylene (3, 4), polypropylene (5, 6) and polystyrene (7), due to their low cost, ease of manufacture, and better mechanical properties.

Meanwhile, the production of petroleum-based packaging materials still increases at 8% per year, with recycling rate <5% (8). Most plastics degrades in incineration and landfill, resulting in the production of microplastics smaller than 5 mm in diameter. High concentrations of microplastics will alter phytoplankton community structure, and therefore affect food chain and entire ecosystems (9, 10).

**Figure 1:**
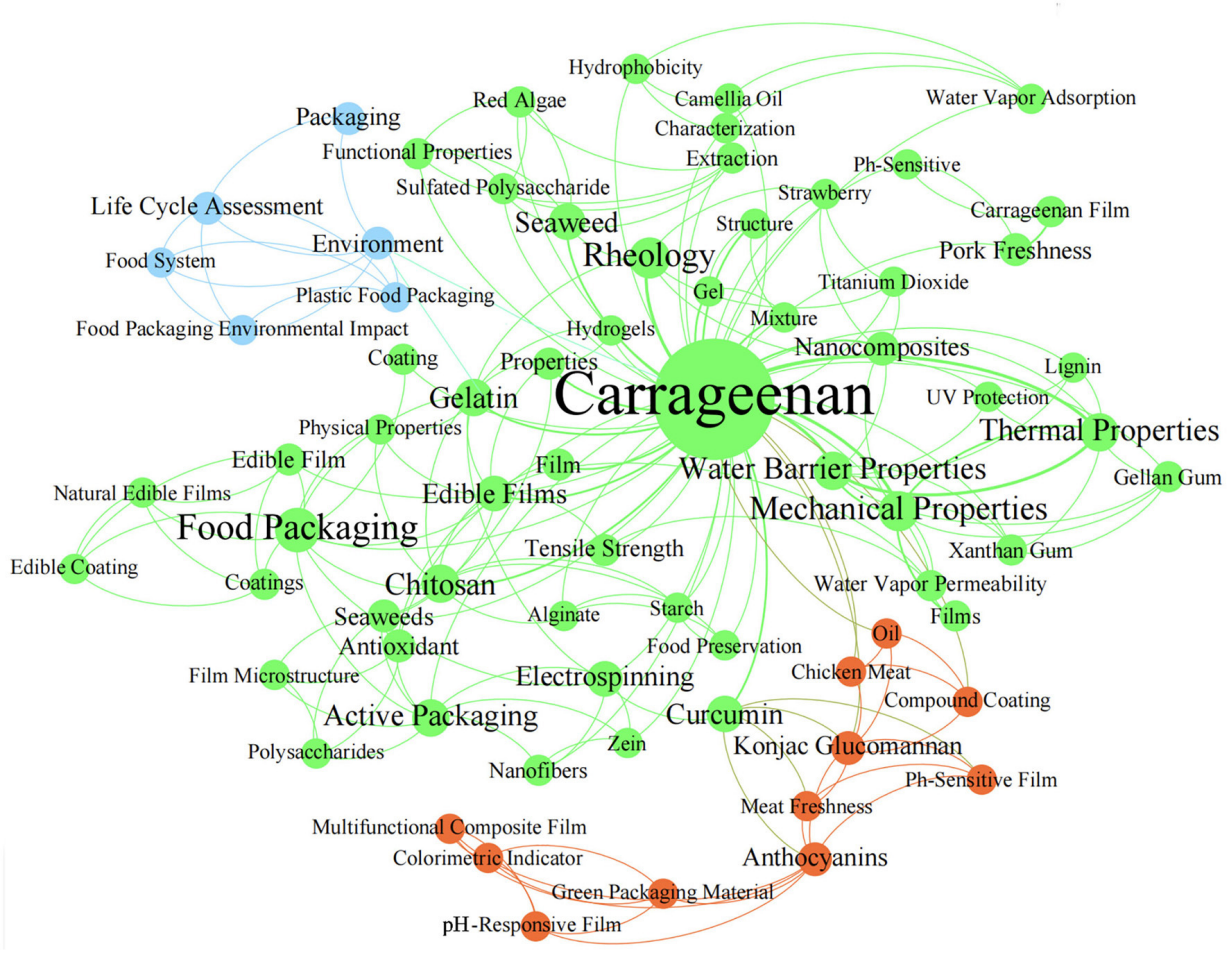
The network visualization of keyword relevance in high-level publications.

With growing awareness of environmental protection, an alternative food packaging material of bio-based polymers has received much attention, because of abundant resource (11), non-toxicity (12), and easily degrade within a few weeks at a specific temperature and humidity (13, 14). Polysaccharides (15–18), lipids (19, 20), and protein (21–24) are usually used as film-forming substrates for biodegradable packaging materials. In this regard, a hydroxyl-riched carrageenan is considered as a highly promising material, due to excellent gelling and film-forming properties. Carrageenan-based composite films are frequently designed by incorporating functional substances, such as polyphenols and enzymes, into carrageenan substrates (25–27). Over the past 2 years, researches focusing on carrageenan-based films have increased sharply. It was found that the number of publications in the research area of food science technology in 2019-2022 was more than five times that before 2019, when keywords like “carrageenan film” or “carrageenan coating” or “carrageenan packaging” were entered into search box of “topic” within scientific database “Web of Science”. However, only one review published in 2020-2022, which focused on the effect of nanomaterials (such as TiO_2_, SiO_2_, and copper sulfide) on carrageenan films, the properties of nanomaterial-enriched carrageenan films and their applications (28). Our study offers an overview of the latest comprehensive development of carrageenan-based biodegradable films for food packaging, focusing on high-level studies over past 3 years (Figure 1).

**Figure 2:**
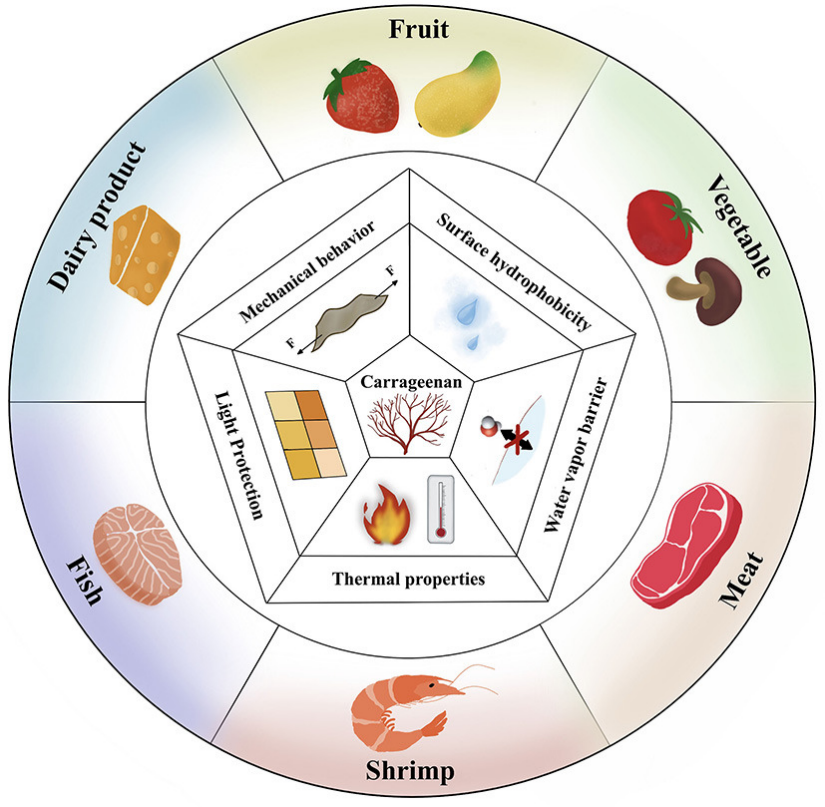
Properties of the carrageenan-based film and its application in food packaging.

In order to discuss this topic logically, the source, extraction methods and characteristics of carrageenan were firstly summarized, and then the carrageenan gelation process and driving forces for the formation of carrageenan-based films are highlighted. Finally, properties and applications of carrageenan-based films were discussed in detail (Figure 2).

## Properties and preparation of carrageenan

**Figure 3:**
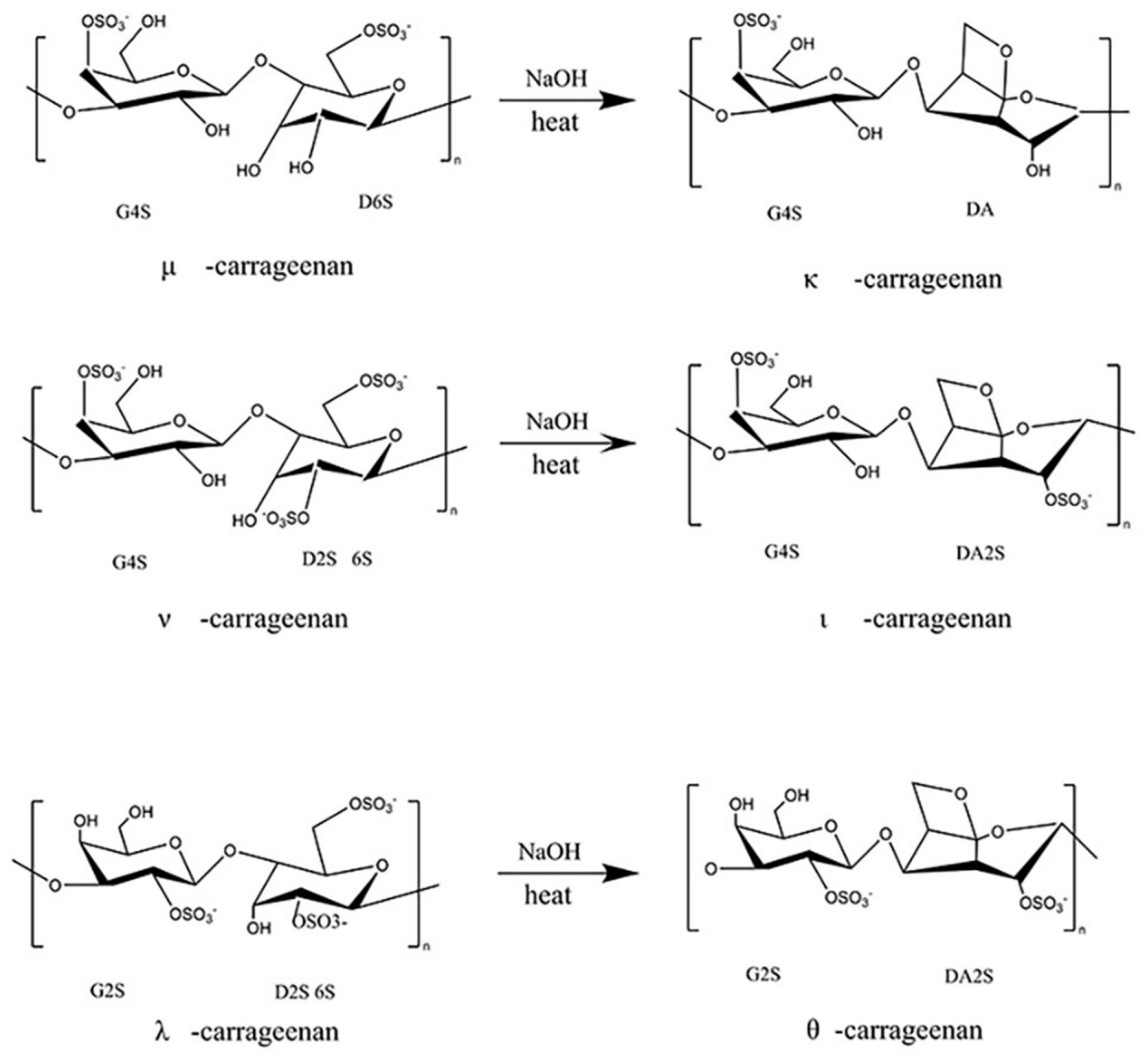
Conversion between different types of carrageenans.

Carrageenan is composed of alternating copolymers of 3,6-anhydrous-galactose (3,6-AG) and D-galactose linked by α-1,3 and β-1,4-glycosidic bond (27, 29, 30). According to different number and position of sulfate groups, carrageenan can be divided into six basic forms, namely kappa (κ)-, lambda (λ)-, Iota (ι)-, Mu (μ)-, Theta (θ)- Nu (ν)- and ξ (xi)- carrageenan (31). With alkaline pretreatment, μ-, ν- and λ-carrageenan can be translated into κ-, ι-, and θ-carrageenan, respectively (Figure 3). Among them, κ-, ι-, and λ- carrageenans are the most widely used commercial products, which have one, two and three sulfate ester groups in each disaccharide repeat unit, respectively (32). In general, κ-carrageenan exhibits the strongest gelling ability because it contains about 25–30% sulfate groups and 28–35% 3, 6-anhydrogalactose contents (30).

All types of carrageenans are insoluble in organic solvents, oils, and fats, but soluble in water. The water solubility of carrageenan is influenced by multiple factors, such as the sulfate group on the molecule and the content of 3, 6-anhydrogalactose (33). Compared with other commercial carrageenans, κ-carrageenan exhibits lower aqueous solubility, because of low sulfate ester groups and high 3, 6-anhydrogalactose contents (34).

Carrageenan is extracted from red algae, such as *Eucheuma Cottonii* (35), *Mastocarpus stellatus* (36), and *Hypnea musciformis* (37), by multiple methods, including hydrolysis, ultrasonic-assisted and microwave-assisted extraction. The hydrolysis extraction is the most frequently used method, and the carrageenan yield can reach 27% (38). However, the extraction steps are complicated, solvent-consuming and time-consuming. In contrast, ultrasonic-assisted and microwave-assisted extraction methods possess shorter extraction time, lower energy consumption, and higher extraction yields, which have drawn an increasing attention. Ultrasonic-assisted extraction is a physical technology that can generate high pressure and high temperature in a short period of time, thereby generating high-intensity shear force, free radicals and shock waves, and resulting in physically destroying the plant cell wall (39, 40). A 50–55% carrageenan yield can be achieved within 15 min at the ultrasound power of 150 W (38). Since algae with high moisture contents are highly susceptible to microwave radiation, the microwave-assisted extraction is also an auxiliary method that can heat sample and solvent rapidly (41). The parameter settings are critical and have a considerable effect on the carrageenan yield. For example, the yield of carrageenan at a microwave temperature of 105 °C is 30.7% higher than that at 85 °C (42). Also, the carrageenan yield under neutral extraction conditions is approximately 37% higher than under alkaline conditions (43). Furthermore, all of these methods could change the structure, bioactivity, and composition of carrageenan. Therefore, the combination of multiple extraction technologies will be future trends in carrageenan extraction methods.

## Film-forming of carrageenan

### The gelation process of carrageenan

**Figure 4:**
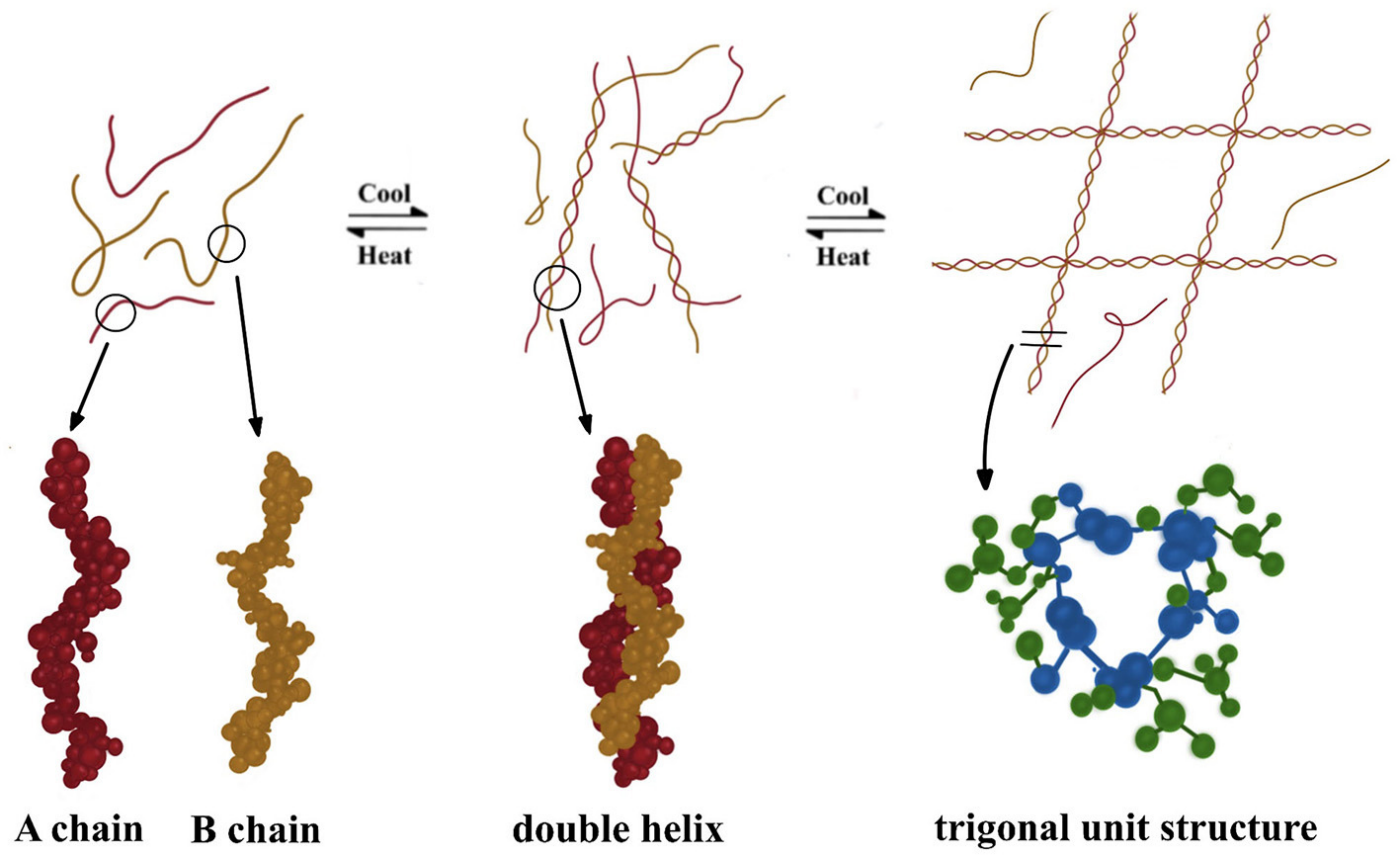
Schematic diagram of the carrageenan film forming process.

The gelation of carrageenan is a complex process, involving the coil-helix transition and then the helix aggregation (Figure 4). In the initial stage, the carrageenan in the sol state mostly exists in the form of irregular coils. Then the low-temperature cooling process induces formation of hydrogen bonds between the galactosyl units, which promotes the twisting of the anhydrogalactose sequence in a helix manner (44–46). As for the helix aggregation stage, double helices arranged laterally in a trigonal unit structure are interconnected by intermolecular interactions to form individual junction region in units of six to 10 molecules (44, 47), thereby resulting in forming cubic structured carrageenan gels (45). The number of molecules in the bonding area is affected by the cooling rate, and a large bonding area with more molecules is likely to be formed at the slower cooling rates (48). The higher the number of molecules in the junction zone, the more rigid the gel is. Therefore, the multi-molecular junction region of κ-carrageenan exhibits rigidity when disturbed by shear force, whereas the ι-carrageenan gel, consisting of only two molecules in the linkage region, has a more flexible structure and is less sensitive to shear (41, 49, 50).

### Main driving forces in the formation of carrageenan-based films

#### Electrostatic interaction

In general, some salt ions, such as K^+^ and Ca^2+^, have been introduced into the film forming process (33). Because salt ions can partially neutralize the negative charge of sulfate groups in the carrageenan ionic chain through electrostatic interactions, thus reducing the electrostatic repulsion between sulfate groups, and facilitating a disorder-order conformational transition (24, 51). Other components have also been added into the carrageenan matrix through electrostatic interactions, such as gelatin and sodium caseinate. Disordered κ-carrageenan combined electrostatically with gelatin to facilitate the gelation process, leading to a high temperature of κ-carrageenan/gelatin conformational transition than that of pure carrageenan in film-forming process (52). In addition, sodium caseinate and negatively charged λ-carrageenan have been proposed to fabricate edible films. The cross-linking of polymer chains formed by electrostatic interactions could reduce the water vapor permeability of the film and improve moisture barrier properties (53).

#### Hydrogen bonds

As one of the most common non-covalent bonds, hydrogen bond is widely distributed in structure of the carrageenan film and critical for formation of pure carrageenan films. It is mainly explained by that the abundant hydroxyl groups in carrageenan molecules can form intramolecular or intermolecular hydrogen bonds with each other. Moreover, other polysaccharides have commonly been added to carrageenan for improving the properties of the resulting films (45, 54, 55). Agar molecules and κ-carrageenan molecules can be intertwined through hydrogen bonds to form agar/κ-carrageenan mixed double helixes (56). Similarly, arrowroot starch molecules and amylose can combine with carrageenan molecules through hydrogen bonding, resulting in films with superior mechanical properties compared with pure carrageenan film (57).

### Fabrication techniques of carrageenan-based films

#### Direct coating

Direct coating is commonly used for fabricating edible packaging, including spray coating and dipping (58–60). Spray coating is a technology in which high pressure is usually used to atomize the film-forming solution and spray it onto the food surface (59, 60). Although the efficiency of spray coating is high, the operation is complicated and requires the professional equipment. Compared with the spray coating method, the dip coating method has been used for a more extensive range of applications. It involved preparation of the coating solution, dipping the food in coating solutions, removing excess coating solutions, and air-drying of the coating (61, 62). As it is known, the coating thickness closely affects films properties, and the coating thickness by dipping method is generally larger than that prepared by spray method (63). The dipping method is relatively simple to operate, but it requires a large amount of film-forming solutions. In summary, since the coating is directly contacted with the food, films prepared by the direct coating method can interfere with the sensory aspects of consumers, which requires the films with acceptable sensory properties.

#### Solvent casting

Solvent casting is the most common method for manufacturing films, which involves preparation of film-forming solutions, spreading film-forming solutions on a suitable mold and drying. Carrageenan/alginate (64), carrageenan/gelatin (65), and carrageenan/starch (66), composite films are usually formed by this method. Numerous factors can affect the film formation process. For instance, the film-forming mold can have a great impact on the peeling of a film, and molds that are easy to peel off films are teflon, glass plates and so on (67). In addition, the drying temperature and the environment humidity affect the film thickness. Specifically, the thickness and elongation at break of the film decreased with an increase in drying temperature (68). It has also been proven that the tensile strength and water vapor permeability of the film increased with increasing ambient humidity (69). Furthermore, the film prepared with high concentration of carrageenan film-forming solutions has been found to possess a poorly sponge structure and large pores, which will be detrimental to the subsequent utilization of the film (70). It may be due to the delayed delamination during film casting. In summary, this method possesses super characteristic of low cost, simple equipment and easy operation, but it require a long drying time, which is not conducive to its application in large-scale commercial production.

#### Electrospinning

Electrospinning is a non-mechanical technology that makes polymer materials into nanoscale fibers. It can generate a high-voltage electrostatic field that is used to change the surface of a polymer solution droplet and apply an electrical potential between the polymer solution droplet and the collector at the end of the capillary (71, 72). When the applied electric field force is sufficient to overcome the surface tension of the droplet, the spinneret will eject a jet of charged polymer solution. During the spraying process, the solution jet is stretched by force, the solvent gradually evaporates, and then a partially or fully solidified electrospun fiber is obtained (72, 73). The final prepared electrospun fibers possess ultra-fine structure, high porosity, high surface-to-volume ratio, and tailored morphology. Also, they can be used to encapsulate bioactive ingredients to improve antimicrobial properties, oxidation resistance (74, 75), and thermal stability (76) of films. For instance, zein, carrageenan, ZnO nanoparticles and rosemary essential oil were used to form maize protein/κ-carrageenan electrospun fibers by electrostatic spinning, and the developed fibers exhibited better surface hydrophobicity, high antibacterial, as well as antioxidant properties (73). Owing to its simple operation and low cost, electrospinning is one of the most effective methods for preparing one-dimensional materials. However, the weak interaction between electrospun fibers endows the films with poor mechanical properties.

#### Extrusion

Owing to fast, high efficiency, high energy utilization rate, and suitable for large-scale production, the extrusion method is commonly used for the production of packaging materials. The material is extruded by a twin-screw, passing through a desired shaped die to fabricate films under certain parameter settings (77). Unlike petrochemical materials, biopolymers are more sensitive to processing parameters such as screw speed, temperature, feed rate and screw configuration (78). It has been demonstrated that high extrusion temperature and unsuitable pH will break the polypeptide chain of the protein and affect the protein charge distribution (79). Also, small changes in extrusion processing parameters will have a considerable impact on film properties. In summary, complex parameter settings have been a challenge for the production of biopolymers-based films by extrusion. At present, the extrusion method has been used to prepare some biopolymers-based films, such as pectin-based and gelatin-based films (78, 80). Studies have shown that uniform biopolymers-based films can be produced by precisely controlling extrusion speed, barrel temperature and pH (81). However, the preparation of the carrageenan films by the extrusion method have rarely been investigated, and only related reports on the preparation of carrageenan hydrogels and particles by the extrusion method (82, 83). Therefore, further attempts requires to be made to fabricate the carrageenan film using the extrusion method to increase commercial viability of the production of carrageenan-based packaging films.

## Physical and chemical properties of carrageenan-based film

Carrageenan is regarded as a promising biomaterial for fabricating food packaging films, due to its unique mechanical behavior, water vapor barrier, surface hydrophobicity, light protection and thermal properties. In this section, it focused on the outstanding properties of carrageenan.

### Mechanical behavior

The mechanical behavior of the film is usually characterized by tensile strength and elongation at break (84). The measurement process is as follows: the film sample is cut into small pieces and then subjected to a tensile test to obtain a stress-strain curve (85). After analyzing the trend of the curve, the average value of each measured characteristic can be obtained (86).

As shown in Table 1, the tensile strength (TS) and elongation at break (EAB) of the pure ι-carrageenan film were 2.5 MPa and 1.04%, respectively (85). In comparison with the pure ι-carrageenan film, due to a low content of sulfate and negative charge, the pure κ-carrageenan film exhibited better mechanical properties, with a TS of 42.5 MPa and an EAB of 3.9%, respectively. However, the neat structure of κ-carrageenan made the pure κ-carrageenan film brittle (99). To overcome the limit of κ-carrageenan, ι-carrageenan was mixed with κ-carrageenan to fabricate the mixed carrageenan film and the TS of the mixed film reached 55.2 MPa (87). It could be explained that anhydro-bridges were formed during the mixing process, which reduced the hydrophilicity of the sugar residues, leading to a conformational shift in carrageenan and enhancing its gelation properties (64).

**Table 1:** The physical and chemical properties of carrageenan-based films.

**Film**	**The physical and chemical properties of carrageenan film**				**References**
	**Mechanical properties**		**Water vapor permeability (WVP)/10^−11^ g·m·Pa^−1^·m^−2^·s^−1^**	**Thermal stability/%**	
	**TS/MPa**	**EAB/%**			
Pure κ-carrageenan	42.5	3.9	7.5	-	(87)
Pure ι-carrageenan	18.36	9.86	36	40.58	(88)
κ/ι-hybrid carrageenan	55.2	3.4	6.7	-	(87)
κ-carrageenan-cellulose nanocrystals	85.29	27.7	-	41.54	(89)
κ-carrageenan/konjac glucomannan/TiO_2_ nanoparticles	63.7	28.8	9.02	18.5	(90)
κ-carrageenan/lignin	27	28	-	47.3	(91)
unidirectionally permeable film (κ-carrageenan/gelatin/curcumin/zein/glycerol)	12.15	12.97	1.69	-	(65)
κ-carrageenan/palm oil/emulsifier	13.83	43.61	15.4	-	(92)
κ-carrageenan/starch	13.6	16.7	9.6	-	(66)
ι-carrageenan/starch-fatty/ stearic acid/palmitic acid/lauric acid/butyric acid/oleic acid	218 (N/m)	-	1.18	-	(93)
Double-layer indicator film (κ-carrageenan/curcumin/anthocyanin/konjac glucomannan/camellia oil)	22.64	52.3	1.85	12.56	(94)
κ-carrageenan/whey protein isolate/pomegranate seed oil	6.18	23.43	3.14	-	(95)
κ-carrageenan/mulberry polyphenolic extract	26.3	8.59	3.86	-	(96)
κ-carrageenan/hydroxypropyl methylcellulose/Prunus maackii pomace	10.78	43.2	2.07	-	(97)
κ-carrageenan/curcumin	18.12	16.99	9.8	-	(98)
κ-carrageenan/xanthan gum/gellan gum hydrogel	20.87	13.7	21.5	43.6	(84)

The incorporation of other components into the carrageenan matrix resulted in changes in crosslinking properties of carrageenan, thus affecting the mechanical properties of the carrageenan-based film (84). For example, it has been demonstrated that with the addition of TiO_2_ nanoparticles increasing from 0 wt to 5 wt%, the tensile strength and modulus of the composite films significantly increased from 39 to 122% (100). It may be explained by that the addition of TiO_2_ nanoparticles can strengthen the physical interaction between the filler and the carrageenan matrix. Better still, sodium benzoate solution was coated onto the film surface as a photosensitizer and then the film was exposed to the UV light for a photo-crosslinking. The reactive benzoate radicals generated by UV radiation reacted with the tertiary hydrogen atoms of the semi-refined carrageenan polymer, forming polymeric radicals, which promoted the crosslinking of the carrageenan polymer chains. The TS of the resulting film was increased by 36–55%, and the density and water barrier properties of the resulting film were also enhanced (101). However, when the addition of nanomaterials is excessively large or the UV exposure time is excessively long, the resulting film will not exhibit excellent mechanical properties, due to the crystallization of nanoparticles into clusters and the pores and microcracks caused by high level photodegradation (100, 102).

In recent years, due to the increasing environmental issues in the world, natural biodegradable ingredients have also been incorporated into the carrageenan matrix. For instance, the pearl millet starch was mixed with carrageenan to form the composite film with excellent mechanical properties (TS of 28 MPa) (103). It may be ascribed to the formation of hydrogen bonding between the starch and carrageenan molecules, resulting in increasing intermolecular interactions (103). Furthermore, the addition of edible maize protein increased the elongation at break of the film from 10.85 to 12.97% (65). In summary, most additives are mixed with carrageenan through physical interactions, and the mechanical properties of carrageenan films are affected by additives (types and amounts). Therefore, natural additives, such as starch, animal and vegetable proteins, are suitable choices due to the widespread presence of hydroxyl groups in their molecular chains and excellent compatibility with carrageenan matrix.

It is noteworthy that plasticizers also affect the mechanical properties of composite films. The addition of plasticizers increases the fluidity of the film-forming matrix by distorting the hydrogen bonds between adjacent polymer chains. However, the addition of plasticizers at high concentration could increase the intermolecular spacing and reduce the tensile strength of the film (103).

### Water vapor barrier

The water vapor barrier of a film is affected by several factors, such as the integrity of the film, the hydrophilic-hydrophobic ratio of the film components, the molecular density of the film and the fluidity of the polymer chains (84). Carrageenan exhibits poor water resistance, which limits its application (104). Therefore, the addition of other ingredients is needed to improve the water vapor permeability of composite films. For example, the cross-linked network formed by zein and carrageenan allows water molecules to pass through a twisted path, thus stopping water vapor outside the food. It has been demonstrated that the addition of zein/carrageenan complex can improve the water vapor permeability of the gelatin film, decreasing from 5.2 ± 0.2 × 10^−10^ g mm/h mm^2^ Pa (the pure gelatin film) to 1.2 ± 0.1 × 10^−10^ g mm/h mm^2^ Pa (the zein/carrageenan/gelatin film) (105). In addition, the hydrogen bonding interaction between the hydroxyl groups on the benzene ring of curcumin and carrageenan made the curcumin evenly distributed in the carrageenan matrix, thus prolonging the permeation path of water vapor, and reducing the water vapor permeability of the film from 2.08 × 10^−10^ g mm ^−2^ s^−1^ atm ^−1^ to 0.98 × 10^−10^ g mm ^−2^ s^−1^ atm ^−1^ (98).

### Surface hydrophobicity

**Figure 5:**
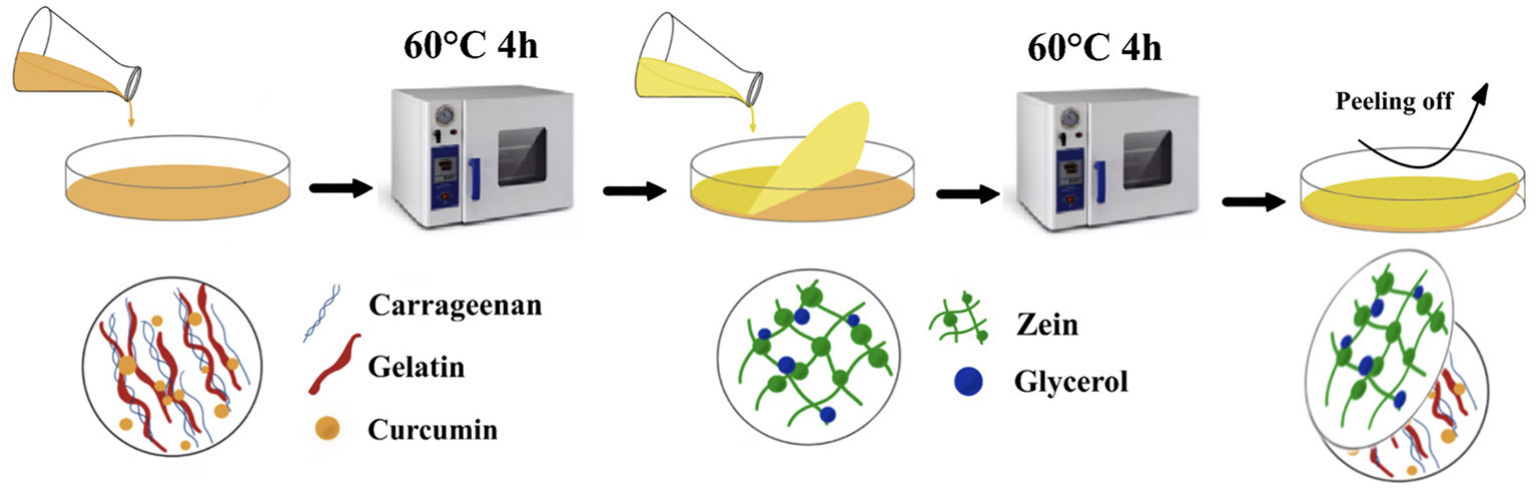
Preparation of the unidirectionally permeable film.

The hydrophobic surface protects the film from moisture and facilitates the removal of impurities by water. However, due to its hydrophilicity, carrageenan film exhibits poor moisture-proof effect, which limits its application in food preservation (92). Therefore, the interest in the incorporation of other components into the carrageenan matrix for enhancing hydrophobicity of the films has been growing. For example, the high pressure treatment broke the intermolecular bonds in the starch molecules, allowing hydrogen bonds to form between the starch and carrageenan. Therefore, the pressure-treated starch was dispersed into the carrageenan matrix, forming a compatible system of carrageenan and modified starch with enhanced surface hydrophobicity and tensile strength (66). The addition of fatty acids also had a positive effect on surface hydrophobicity. Compared to the control films without fatty acids, polymer-lipid-carrageenan composite films showed better surface hydrophobicity, due to the generation of strong stabilizing network linked by covalent and non-covalent bonds (93). Proteins were used to mix with κ-carrageenan, and exposed its internal hydrophobic groups, thus improving the hydrophobic properties of the composite films (99). In addition, some small molecules, such as arginine laurate, salt ions can improve the hydrophobicity of carrageenan films (106). The surface activity of the hydrophilic chain of carrageenan was successfully enhanced by the in *situ* interaction of the polymer with the surfactant arginine laurate. Furthermore, a unidirectionally permeable film was prepared, including an inner layer (a carrageenan film) and an outer layer (a hydrophobic film) (Figure 5), which is beneficial to improve the hydrophobicity of the composite film (65).

### Light protection

**Figure 6:**
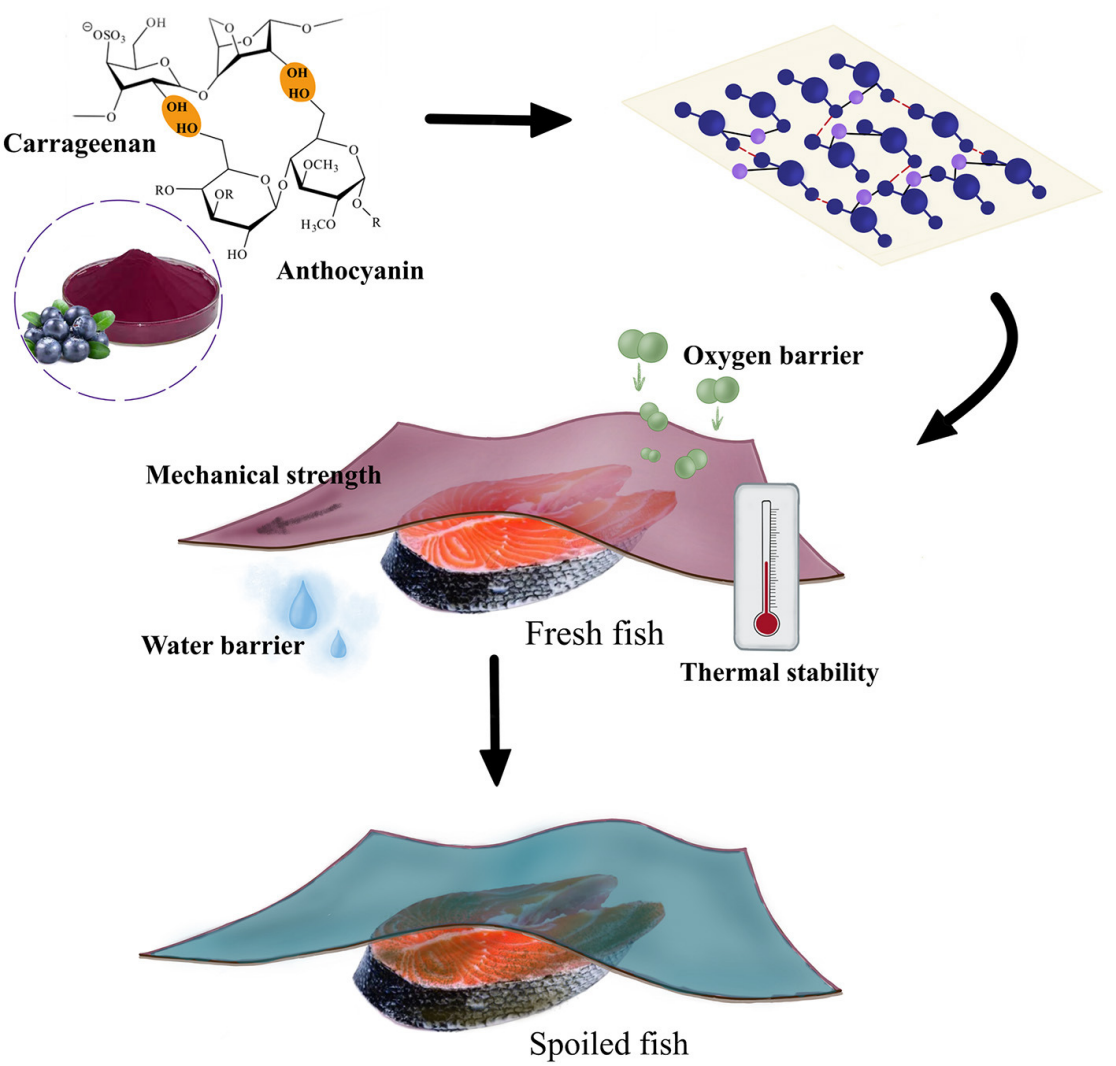
Schematic diagram of the application of the anthocyanin-carrageenan film for monitoring fish freshness.

The light barrier properties of carrageenan films are vital for food packaging applications. There are two properties of the film to achieve light protection for food, including color and opacity. The color of the carrageenan composite films is measured using a colorimeter and evaluated by the L, a and b values (112). L is for lightness and darkness, ranging from 0 to 100. As for a and b values, they range between−128 and + 127. Specifically, a value represents red (+127)/green (-128), and b value is yellow (+127) /blue (-128). The pure carrageenan film is nearly colorless and transparent, and researchers often enhance the reflection of light by increasing the whiteness of the film to protect food. For example, due to the aggregation and mutual cross-linking of white fibers, the addition of soybean dietary fibers improved the whiteness of the carrageenan film, thus protecting food (107). The addition of colored substances endows the film with color, thus protecting food. For instance, the addition of phenolic extracts (from germinating fenugreek seeds) changed the color of the carrageenan composite films. Specifically, the L value of the film decreased from 91.51 to 66.87, and the color became significantly darker, which can enhance the UV-resistance of the films (108, 109). Curcumin and anthocyanin are often added into carrageenan composite films, which can improve the light barrier properties of the composite films. For example, with the curcumin content of the carrageenan film increasing from 0 to 7%, a value of the film showed an increase from−1.08 to + 21.88, b value of the film increasing from +4.82 to +65.29, L value of the film decreasing from 96.16 to 61.88. The color of the film darkened and deepened in yellow (98). Furthermore, since the color of curcumin and anthocyanin changes with pH, the pigment-loaded carrageenan composite film can be used as an intelligent colorimetric indicator film (110, 111) (Figure 6).

The opacity of the film can affect food protection of the film. The addition of nanoparticles made the film cloudy, increased the opacity of the film, and prevent light from passing through (112). Similarly, the incorporation of oil droplets into the film can cause light scattering, and the opacity of the film was affected by many factors, such as the content of the oil droplet, the size of the oil droplet, and the light scattering intensity (113). For instance, the light transmittance of the carrageenan film (containing camellia oil) at 350 nm was lower than 20%, while that of the pure carrageenan film was about 60%. It indicated that the addition of camellia oil can enhance UV-resistance and light-resistance of the films (94).

### Thermal properties

The thermal properties of carrageenan films determine the ability of the film to adapt to the environment temperature. Thermal properties are usually determined using thermogravimetric analysis and differential scanning calorimetry (90, 114). Thermogravimetric analysis involves weighing the film into a crucible, scanning it at an appropriate rate over a range of temperatures, and plotting the mass loss of the sample as a function of temperature (90). Differential scanning calorimetry is plotted as a function of heat flow rate and temperature (115).

In the film-forming process, the addition of the plasticizer causes the carrageenan chains to be separated and the intermolecular interaction forces to be reduced, thus affecting the thermal property of the film. For example, the glass transition temperature of the carrageenan films (with plasticizers) dropped below 30 °C, while the glass transition temperature of pure κ-carrageenan was 84.72 °C (35). Therefore, the selection of the plasticizer is critical for thermal properties of the film. Glycerol and sorbitol are commonly used as plasticizers in the film-forming process and have different effect on the thermal performance of the film. It was found that the melting temperature of the film decreased sharply with increasing glycerin concentration, while the melting temperature of the film did not decrease as sharply with increasing sorbitol concentration (35). Moreover, the film (with glycerin) still exhibited good stability at around 200 °C, which can meet the needs of most food packaging (35, 116).

In order to endow carrageenan films with a wide range of applications, the incorporation of substances that enhance the internal structural stability of the carrageenan, can improve thermal properties of the composite film. Transglutaminase, an enzyme that occurring almost all organisms, can significantly enhance the double helix structure of carrageenan through crosslinking. As the concentration of transglutaminase increased, the melting temperature of the film increased from 49.7 °C to above 80 °C (117). In addition, the incorporation of the natural peptide ε-polylysine can exhibit excellent compatibility with carrageenan and promote the formation of the ordered and tightly arranged network structure in the carrageenan gel, thus improving the thermal stability of the carrageenan film. It was proven that the decomposition temperature of the film (with ε-polylysine) was much higher than that of the pure carrageenan film (118).

## Application of carrageen-based films on food preservation

### Applications in fruits and vegetables

Fruits and vegetables are rich in vitamins and minerals, which play an important role in human health. However, they are susceptible to spoilage. Approximately 20% of fruits and vegetables are discarded each year in developed countries, thus leading to huge economic losses (119). Generally, main factors affecting the spoilage of fruits and vegetables are water content and respiration rate. It has been proven that an extremely high water content (about 75–95%) and high respiration rate endowed fruits and vegetables susceptible to bacteria and fungi, therefore resulting in spoilage (120). So, it is necessary to develop preservation methods for extending the shelf life of fruits and vegetables.

The carrageenan film has excellent mechanical and protective properties, therefore it is favored in the preservation packaging of fresh fruits and vegetables (Table 2). The mechanical properties of the carrageenan film allow the film to maintain intact during the transport and distribution of fruits and vegetables, thus avoiding mechanical damage to the products (123). Furthermore, the addition of oxygen and ethylene scavengers into the carrageenan film can slow down the respiration rate of fruits and vegetables and inhibit the growth of aerobic bacteria (125). Although carrageenan exhibits a weak water vapor barrier (104), the addition of other components could effectively reduce the water vapor permeability of the film, thus preventing the loss of internal moisture in fruits and vegetables through pores and cracks on the surface (123).

**Table 2:** Application of carrageenan-based packaging.

**Fruits or vegetables**	**Carrageenan**	**Additives**	**Preservation behaviors of carrageenan-based films**	**References**
Strawberry	carrageenan	Lemon grass essential oil	Shelf-life was extended up to 12 days	(62)
Strawberry	κ-carrageenan	Sodium carboxymethyl starch, carboxylated cellulose nanocrystals	The film keeps the inside of the strawberry fruit moist after a week of storage	(121)
Mongo	κ-carrageenan	ZnO nanoparticles	The coating can reduce the amount of O_2_ and limit the diffusion of CO_2_ out of the tissue. On the 33^rd^ day of storage, the degree of deterioration of mangoes was still very low	(122)
Plum	ι-carrageenan	Rice starch, sucrose fatty acid esters	The respiration rate of the experimental group was lower than that of the control fruit, and the coated plums stored at room temperature for three weeks remained firm and had good color	(123)
Tomato	ι-carrageenan	Arrowroot starch	The coating reduced the weight loss of tomatoes at room temperature and extended their shelf life up to 10 days	(57)
Mushroom	carrageenan	Nano-SiO_2_, konjac glucomannan	The addition of Nano-SiO_2_ reduced the gas permeability of the coating and delayed the effect of UV light on food quality	(124)

**Figure 7:**
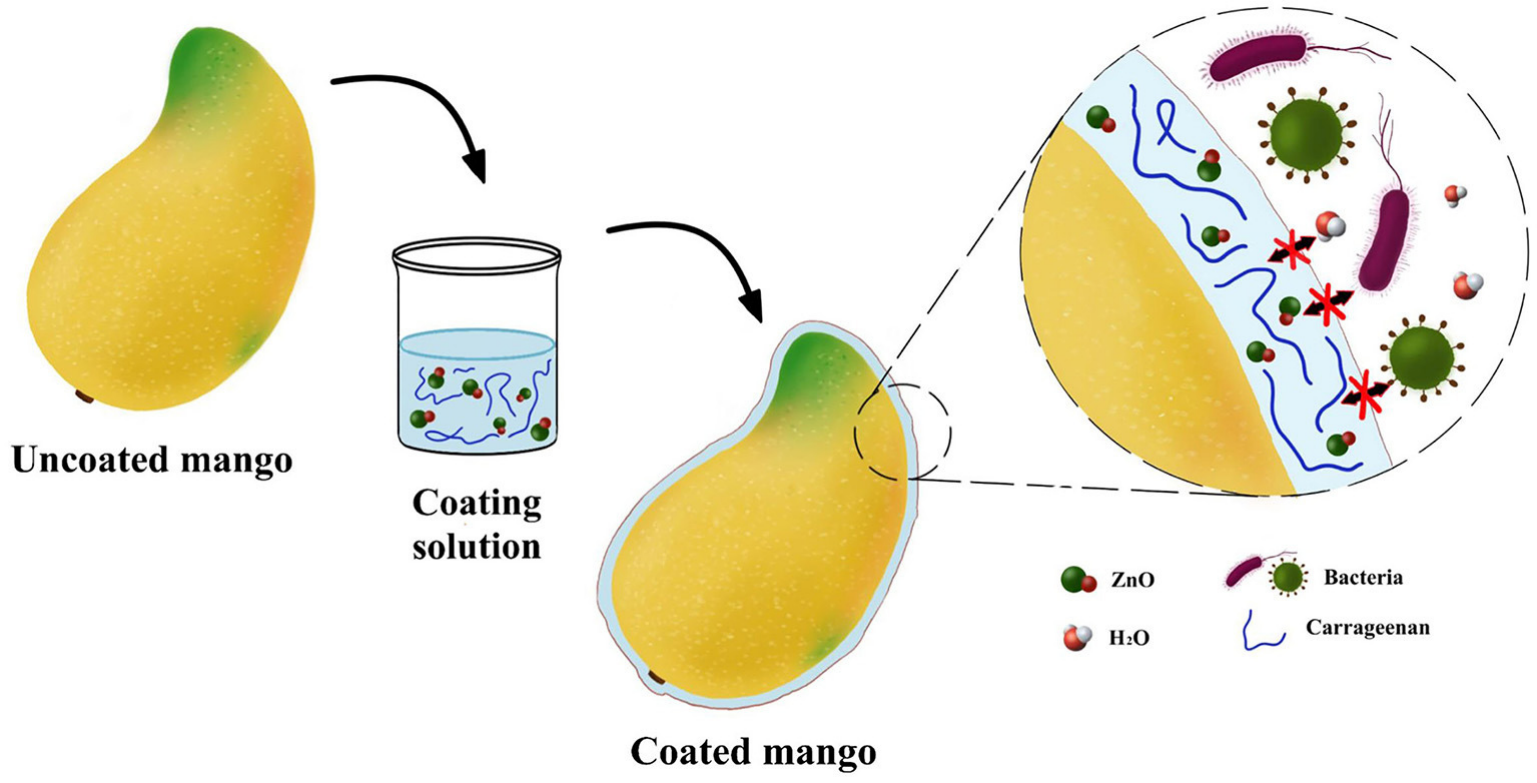
Schematic diagram of the application of the carrageenan-ZnO nanocomposite coating for mango preservation.

It has demonstrated that carrageenan-zinc oxide nanocomposite coatings (Figure 7) can reduce physical and biological damage to mangoes (122). On the 19^th^ day, coated mangoes still retained initial firmness, a slight discoloration and decay occurred in the coated mangoes on the 33^rd^ day. The shelf life of coated mangoes was extended to 14 days, compared with uncoated mangoes (122). Because the addition of zinc oxide nanoparticles allowed the composite coating to significantly increase the tensile strength by 43%, reduce the water vapor transmission by 9%, and inhibit the growth of *E. coli*, compared with that of pure carrageenan coatings (122). Compared with the mango, strawberry is more prone to spoilage. Therefore, it is imperative to develop a method for strawberry preservation. The carrageenan coating has been used for strawberry packaging, and exhibited the lowest total soluble solids (9.50%) on the 12^th^ day of storage, much lower than the control group (12.20%) (62). The rot rate of strawberries was reduced by 14.29% since the carrageenan film inhibited the metabolic activity and reduced water loss (62).

Carrageenan film can play a similar role during vegetable storage. Tomatoes coated with a naturally biodegradable edible film (arrowroot starch and ι-carrageenan) have been demonstrated to be more firm than unwrapped tomatoes (57). Konjac glucomannan-SiO_2_-carrageenan composite nano films can also reduce the weight loss and oxygen permeability of white mushrooms, thereby slowing down the respiration of mushrooms and reducing their browning index, extending the shelf life of mushrooms stored at 4 °C by 5-12 days (124).

### Applications in fish and meat products

Fish and meat products are favored for their high protein and unsaturated fatty acid content (65). However, fish and meat products are prone to spoilage. The spoilage of fish and meat products is affected by microbial contamination, fat oxidation, protein degradation and endogenous enzymes (126). In addition, environmental factors such as temperature, oxygen, moisture and light, can easily cause changes in the color, smell, texture and flavor of fish and meat products, which are difficult to detect with the naked eyes (127, 128). Low temperature preservation is commonly used for preservation of fish and meat products, including refrigeration and freezing. However, frozen fish and meat products do not taste good, and cold fish and meat products are more popular. Therefore, in order to extend the shelf life of the cold fish and meat products, the development of carrageenan-based composite films has received tremendous attention. For example, compared with unpackaged beef, the application of copper sulfide nanoparticle-carrageenan films to beef can reduce effectively the amounts of *Escherichia coli* and *Staphylococcus aureus* by 52.6% and 69.8%, respectively (129). Similarly, it was found that the use of carrageenan/camellia oil films for chicken preservation reduced the amounts of cryophilic bacteria (3.86 Log CFU/g) sharply, compared with that of the control group (5.35 Log CFU/g) (130). In addition, due to the antioxidant and antibacterial activities of curcumin, the carrageenan/curcumin films can inhibit the growth of *Staphylococcus aureus* and *Serratia marcescens*, thus being applied for shrimp packaging (98).

Spoilage of fish and meat products is often accompanied by the production of nitrogenous substances such as ammonia, dimethylammonium and trimethylamine, which may alter pH (98). Therefore, the incorporation of pH-sensitive substances into carrageenan matrix allows the composite films to monitor the freshness of fish and meat products. For example, the curcumin-loaded carrageenan film has been developed for monitoring pork freshness, since the phenolic hydroxyl groups of curcumin readily reacted with OH- to form phenolate anions (98). It was found that the color of the film changed from yellow to red, as the TVBN value of the pork increased from 4.91 to 31.11 mg/100 g (108). The κ-carrageenan/anthocyanins films can also be used as an indicator film for monitoring pork freshness. Studies have demonstrated that the color of the indicator film turned from purple to green, with the TVBN value increasing from 8.23 to 14.63 mg/100 g (131). In lard packaging, the color of the carrageenan/carboxymethylcellulose/plum sap composite film changed from deep red to blue-gray, with the pH increasing from 3 to 13 (132). The natural dye-loaded carrageenan composite films are also applied for fish (112) and shrimp (98) preservation.

### Applications in dairy products

Oxidation and microorganisms are important factors affecting the spoilage of dairy products (121). Currently, carrageenan-based composite films have been gradually popularized in the application of dairy product preservation. Studies have shown that the addition of mulberry polyphenolic extract into carrageenan matrix can be applied for monitoring milk freshness (96, 133). It was found that the color of the carrageenan composite film changed from gray, purple to dark pink, when the film was covered on milk stored at 40 °C for 0–6 h (96, 133). Because the lactic acid produced by microbial metabolism increased, the acidity increased, the pH decreased, and thus leading to the color change of the composite film (96).

Furthermore, due to whipping, the oxidation of lipids and the reproduction of microorganisms are accelerated during the production of ice cream, it is imperative to fabricate carrageenan-based composite films for ice cream preservation (134). The aloe vera gel/carrageenan composite film was found to inhibit the reproduction of various microbial pathogens such as *Staphylococcus aureus, Escherichia coli, Streptococcus agalactiae*, and *Klebsiella pneumoniae* in ice cream (135). The carrageenan-based composite film has also been used in cheese preservation. It was proven that the carrageenan-black bean extract film exhibited excellent antioxidant, mechanical and water barrier properties and was coated on the cheese. Results showed that the peroxide value of the coated sample after 20 days storage was 1.25 mEq O_2_ /Kg, while that of the uncoated cheese sample was 4.2 mEq O_2_ /Kg (136, 137).

## Conclusion and future trends

An increasing attention in carrageenan-based films for food packaging has been drawn, due to abundant resource, biodegradability and excellent compatibility of carrageenan. This review provided an overview of the carrageenan (source, extraction method, and property), film-forming methods (direct coating, solvent casting and electrospinning), the property of the carrageenan-based composite film and applications. We focused on the driving forces for the formation of carrageenan-based films and how to enhance the properties of carrageenan-based films. In summary, most of researches focused on the applications of carrageenan-based films in the laboratory, further studies concentrating on industrial scale productions requires to be done. Moreover, the future trend of carrageenan-based films for food packaging applications is to fabricate excellent multifunctional films, the interaction mechanism of carrageenan with other components therefore requires further discussed.

## Author contributions

CC and SC contributed to the writing. MZhu, MZhou, and TC reviewed and revised the manuscript. YH designed the study and wrote the manuscript. All authors contributed to the article and approved the submitted version.

## Funding

This work was supported by Hubei Provincial Natural Science Foundation of China (2020CFB314), China Postdoctoral Science Foundation (2019M662659), the Postdoctoral Innovative Research Position of Hubei Province, the Fundamental Research Funds for the Central Universities (2042021kf0042), the fund of the Beijing Engineering and Technology Research Center of Food Additives, Beijing Technology; Business University (BTBU), China Postdoctoral Science Foundation (2022M712473), the Fundamental Research Funds for the Central Universities (11041910103), and the Students Research Fund (2022264).

## Conflict of interest

The authors declare that the research was conducted in the absence of any commercial or financial relationships that could be construed as a potential conflict of interest.

## Publisher's note

All claims expressed in this article are solely those of the authors and do not necessarily represent those of their affiliated organizations, or those of the publisher, the editors and the reviewers. Any product that may be evaluated in this article, or claim that may be made by its manufacturer, is not guaranteed or endorsed by the publisher.
